# Qsarna: An Online Tool for Smart Chemical Space Navigation in Drug Design

**DOI:** 10.1021/acs.jcim.5c00720

**Published:** 2025-07-30

**Authors:** Marcin Cieślak, Jan Łęski, Olga Krzysztyńska-Kuleta, Justyna Kalinowska-Tłuścik, Tomasz Danel

**Affiliations:** † Chemistry Department, 318683 Selvita, Kraków 30-394, Poland; ‡ Doctoral School of Exact and Natural Sciences, 37799 Jagiellonian University, Kraków 30-348, Poland; § Faculty of Chemistry, Jagiellonian University, Kraków 30-387, Poland; ∥ Cell and Molecular Biology Department, Selvita, Kraków 30-394, Poland

## Abstract

Drug discovery is a lengthy and resource-intensive process that requires innovative computational techniques to expedite the transition from laboratory research to life-saving medications. Here, we introduce Qsarna, a comprehensive online platform that combines machine learning for activity prediction with traditional molecular docking to streamline virtual screening workflows. Our platform employs a fragment-based generative model, enabling the exploration of novel chemical spaces with the desired pharmacophoric features. Users can share results with others, and docking poses can be examined directly within the platform. In our case study, we successfully identified three new hits for monoamine oxidase B with nanomolar potency, which were later confirmed by experimental assays. The user-friendly web interface requires minimal computational expertise, making advanced virtual screening accessible to scientists regardless of their main field of study. Qsarna represents a significant advancement in computational drug discovery by seamlessly integrating complementary in silico approaches and democratizing access to advanced virtual screening technologies.

## Graphical abstract

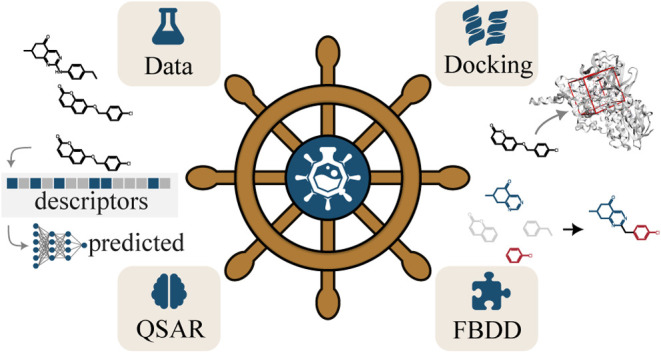

## Introduction

The discovery and development of new therapeutic agents remains one of the most challenging endeavors in biomedical sciences, with estimated costs exceeding $2.5 billion per approved drug and timelines extending beyond 10–15 years.^1^ The significant investment of time and resources, combined with high attrition rates in clinical trials, has prompted pharmaceutical companies and research institutions to seek more efficient drug discovery methods.^2,3^ In this context, computational methods have become essential tools, enabling the rapid evaluation of millions of compounds and the prioritization of the most promising candidates for experimental testing. Virtual screening, in particular, has revolutionized early-stage drug discovery by enabling researchers to systematically assess large chemical spaces and identify compounds with desired properties before initiating costly experimental work.^4,5^ The strategic implementation of computational screening methods early in the drug discovery process has been shown to reduce the number of compounds requiring experimental evaluation by a substantial margin, leading to significant cost savings and accelerated development timelines while maintaining or improving the quality of identified lead compounds.^3,6^

Drug discovery paradigms have evolved significantly over the past few decades, with virtual screening approaches transitioning from simple physicochemical filters to sophisticated machine learning models capable of capturing complex structure–activity relationships. Quantitative structure–activity relationship (QSAR) models, first developed in the 1960s, laid the foundation for modern computational drug discovery by establishing mathematical relationships between molecular structures and biological activity.^7^ Today, advanced machine learning algorithms have surpassed these traditional methods, enabling the autonomous identification of relevant features in molecular structures and uncovering complex patterns from extensive chemical data sets. Recent breakthroughs in deep learning architectures, such as graph neural networks^8^ and graph transformer models,^9,10^ have demonstrated unprecedented accuracy in predicting drug–target interactions,^11^ ADMET properties,^12^ and potential toxicity risks.^13,14^ Furthermore, the emergence of generative models has opened new avenues in drug discovery by enabling the de novo design of molecules with desired properties.^15^ These AI-powered approaches can explore vast regions of chemical space that are inaccessible to traditional screening methods, generating novel molecular structures that maintain synthetic accessibility while optimizing multiple biological and physicochemical properties simultaneously.^16–18^ The integration of multiple screening strategies, combining structure-based methods like molecular docking with ligand-based approaches such as similarity searching and both predictive and generative machine learning models, has become the gold standard in modern virtual screening campaigns.^19,20^ This synergistic approach leverages the strengths of each method while compensating for their individual limitations, resulting in more robust and reliable predictions for candidate compounds and the generation of promising novel chemical entities.

Despite significant advances in virtual screening methodologies, several critical challenges continue to impede their widespread adoption in drug discovery programs. Several AI-driven platforms have been developed in recent years to streamline early-stage drug discovery. Chemistry42 ^21^ is an AI-driven platform combining over 40 generative models with ligand- and structure-based design workflows, offering fine control over molecular properties and synthesis feasibility. DrugFlow^22^ provides an integrated, user-friendly environment for docking, QSAR, ADMET prediction, and virtual screening, tailored for ease of use by nonexperts. MolProphet^23^ is a web-based platform focused on accessible AI-powered drug discovery, featuring pocket prediction, molecule generation from purchasable building blocks, and synthesis planning. Unfortunately, most of the tools described here are commercial and available only through licensing, which can limit accessibility for academic users.

While commercial software solutions exist, there is a notable scarcity of freely available, comprehensive virtual screening platforms accessible to the broader scientific community. Most existing tools focus on single aspects of virtual screening, such as molecular docking or QSAR predictions, but fail to integrate multiple complementary approaches in a unified workflow. This siloed approach often forces researchers to manually combine results from different tools, potentially missing valuable insights that could emerge from a more integrated analysis. Although several online platforms for molecular docking,^24–28^ QSAR modeling,^29,30^ and ADMET prediction^31–34^ exist, and mobile applications like MedChem Game^35^ have demonstrated the potential for accessible drug design tools, none of these solutions provides a comprehensive, guided workflow that combines classical structure-based methods with modern machine learning approaches. This limitation is particularly problematic for medicinal chemists, who, despite their expertise in synthetic chemistry and drug design, may lack the computational background required to effectively utilize these tools. The existing software solutions often demand significant expertise in computational chemistry, including understanding complex parameter settings, file format conversions, and result interpretation. Furthermore, the absence of clear guidance through the virtual screening process can lead to the suboptimal use of these tools, potentially missing promising drug candidates or generating unreliable predictions. The need for a user-friendly integrated platform that combines the power of AI models with traditional virtual screening approaches while guiding users through the process remains largely unmet in the field.

To address critical gaps in virtual screening accessibility and integration, we present Qsarna (**QSAR na**vigator), a web-based platform combining machine learning with structure-based approaches. Our platform provides end-to-end support for virtual screening campaigns, from initial compound library management to candidate selection while maintaining a user-friendly interface for researchers across the drug discovery pipeline. By providing these tools freely to academic researchers through a web interface, Qsarna eliminates the need for local computing resources and democratizes access to advanced methodologies. Our case study on monoamine oxidase inhibitors demonstrates improved success in identifying promising drug candidates compared to single-method approaches. We believe that Qsarna will accelerate drug discovery by enabling the efficient identification of lead compounds.

## Software

Qsarna is an online platform for the automated virtual screening of compounds, seamlessly combining traditional molecular docking software, QSAR machine learning models, and fragment-based generative design. The sections below describe the user-friendly interface design that allows nonexpert users to perform virtual screening experiments effortlessly.

### Graphical User Interface

The graphical user interface was designed to be intuitive. We prepared interactive onboarding tutorials to help new users familiarize themselves with all of the functionalities. We also provide a PDF manual describing all of the modules in detail. Upon registration, an example project is created so that users explore the possibilities of the application without the need to upload their own compounds and prepare screening pipelines. The work on virtual screening campaigns starts with the creation of a new project. Multiple users can be invited to collaborate on adding compound libraries, analyzing screening results, and running additional experiments. All of these functions are accessible through the main project dashboard, presented as a grid of tiles (Figure 1). Screenshots of the most important modules in Qsarna are presented in Supporting Information F.

**Figure 1:**
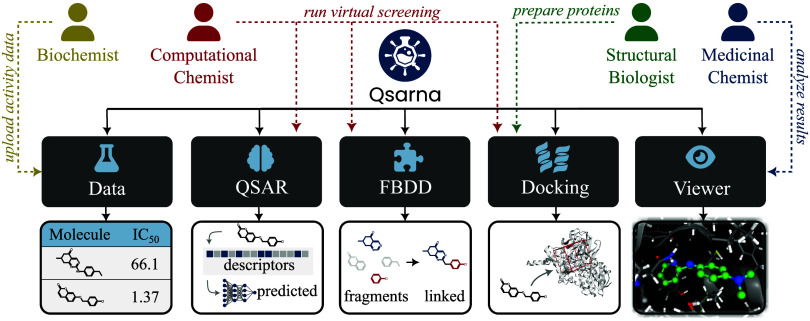
An overview of the Qsarna functionalities. Qsarna allows users to create collaborative projects. The project dashboard displays a grid of tiles similar to those shown here. Data modules enable the uploading of compounds’ structures and activity measurement results. Virtual screening identifies potent molecules using QSAR models and molecular docking simulation with uploaded compounds or automatically linked fragments. Results are visualized to show ligand–target interactions.

### Molecular Visualization

The work with protein structures is facilitated in Qsarna. When new protein structures are uploaded and configured for docking, the chosen bounding box for the running pose search is shown in real time. The results of the screening campaigns can be visualized directly in the application by using the viewer module. Multiple poses of docked ligands can be viewed, and the closest amino acids and ligand–protein interactions are automatically displayed. After the visual inspection, the user can mark the selected poses and assign them a grade on a 5-point scale. Additionally, we provide a molecular dynamics visualization module in which users can upload their molecular dynamics simulation results and inspect the ligand interaction as a function of time.

### Data Management

In Qsarna, all data are managed in a relational database, which is regularly backed up. To ensure data integrity, we employ ACID compliance (atomicity, consistency, isolation, and durability), which means that either the whole transaction is committed to the database or the changes are rolled back. The compounds uploaded by users are automatically preprocessed and deduplicated. If compounds already exist in the user’s project, the records are merged to assign all activity information to one molecular structure. Additionally, chemical libraries can be shared with other users, who can leave comments on the compounds.

### Software Architecture

Qsarna is a Django application implemented in Python 3. The user data including all uploaded compound libraries and analysis results are stored in a PostgreSQL database that is backed up regularly. Unstructured molecular data such as docking poses and protein structures are saved as SDF and PDB files. Similarly, trained machine learning models are stored in a binary file format. All computationally exhaustive tasks are handled by a Celery queue, and the status of each task can be viewed on the task queue page.

Our application supports flexible deployment options ranging from local installations to enterprise-scale cloud infrastructure. The application can be deployed locally using a preconfigured Docker container that ensures consistent environments across different systems. Our cloud implementation leverages Amazon Web Services (AWS). Data integrity and availability are ensured through Amazon RDS, which provides automated backup capabilities and read replicas for uninterrupted database access during high-demand periods. Sensitive research data benefit from server-side encryption via Amazon S3 storage. While the main application runs on Amazon EC2 instances, computationally intensive tasks are automatically distributed across dedicated processing nodes using Amazon SQS for job queuing and Amazon Batch for managed compute environments. This design enables the system to dynamically scale resources based on workload demands. Detailed architectural diagrams for both deployment configurations are provided in Supporting Information E.

## Methods

Qsarna provides three primary tools for hit identification. The first is molecular docking, a traditional virtual screening method. The second and third are machine learning techniques: QSAR models that enhance the quality of nominated hits based on activity data and fragment-based generative modeling, which allows users to expand beyond known molecules. The combination of these tools improves screening reliability by applying orthogonal filters. By training QSAR models on docking results or experimental activity data, the prioritization of docked molecules can be improved, reducing false positives and uncovering nonobvious structure–activity relationships. The generative fragment-linking module explores chemical space creatively and targets parts of the receptor that may be missed by traditional ligand-based screening.

### Molecular Docking

Molecular docking is a structure-based virtual screening method. Qsarna utilizes Smina,^36^ a docking software based on AutoDock Vina.^37^ While defining a new docking protocol, users can adjust docking parameters, such as the exhaustiveness of the pose search, the number of generated poses, and the number of tautomers to be docked. Additionally, users can specify the binding pocket within the application using a protein visualizer that displays the docking bounding box in real time. All ligands are automatically prepared prior to molecular docking, which includes calculating their protonation states (OpenBabel^38^), tautomers, stereoisomers, and low-energy conformations (RDKit^39^). The virtual screening module in Qsarna was tested against three diverse molecular targets using decoy compounds from the DUD-E database.^40^ The results showed that Qsarna performed comparably to Glide^41^ (commercial docking software) on two of the three targets when identifying active molecules (detailed results in Supporting Information C).

### Ligand-Based Activity Prediction

Libraries can also be searched for new active compounds using machine learning QSAR models, following the methodology from the study of Cieślak et al.^42^ The AutoML^43^ tool integrated into Qsarna builds three types of machine learning models: random forests, support vector machines, and artificial neural networks. These models utilize either Morgan fingerprints,^44^ Avalon fingerprints,^45^ MACCS keys,^46^ or RDKit molecular descriptors.^39^ After selecting a set of compounds (minimum 100 for reliable model training) with experimental or computed labels, such as IC_50_ values or docking scores, the data set is automatically constructed and divided into training, validation, and testing sets. The models are automatically trained on one or more data splits while searching for the optimal set of hyperparameters. Models can be trained to predict continuous activity measurements (e.g., IC_50_) or binary activity classes defined by a threshold. Finally, the trained models can be displayed along with their performance evaluations. These models become accessible in the virtual screening module, enabling ligand-based virtual screening of large, unannotated compound libraries. For extremely large libraries, training QSAR models to approximate docking scores provides a rapid alternative to traditional docking-based screening, where only a subset of the original data needs to be docked and used for ML model training.

For all projects, we also provide ADMET predictors for a variety of molecular properties, including the blood–brain barrier permeability,^47^ hERG binding,^48^ CACO-2 permeability,^49^ bioavailability,^50^ and logD. All of these models were trained on the public data sourced from the Therapeutics Data Commons^51^ initiative using the same machine learning models and descriptors available in Qsarna. These properties are automatically predicted for all newly added compounds, facilitating lead identification and multiparametric optimization. Our ADMET models provide comparable results with other publicly available web-based software, such as ADMETlab^52^ and admetSAR^53^ (the evaluation details can be found in Supporting Information D).

### Fragment-Based Design

The drug-like chemical space is extensive, making it often insufficient to depend solely on existing virtual libraries to identify the best binders for a particular target. This is particularly important in identifying novel chemotypes and in avoiding already exploited or patented structures. Consequently, Qsarna offers a generative module that facilitates the creation of entirely new molecules. This module follows the principles of fragment-based drug discovery, allowing users to connect to or expand upon fragments, which are small structures known for their binding capabilities. Our model, CRET,^54^ links these fragments using only known linkers sourced from public databases, enhancing the chances that the resulting molecule can be synthesized. By utilizing experimentally determined or docked fragments, our tool explores the surrounding chemical space to develop focused libraries with potential new candidates. What is important is that the fragment linking exploits the position of the fragments within the binding pocket, ensuring that crucial interactions are preserved. Next, the generated molecules can be redocked using our docking module, prioritizing poses where the fragments are positioned near their initial location. CRET enables the creation of unique and diverse libraries of synthesizable compounds, as confirmed by a benchmark shown in Supporting Information G. This way, users can expand the chemical space around weakly active fragments and create focused libraries of novel compounds that are not found in public compound databases.

## Case Study

We validated our virtual screening software’s capabilities by following the protocol outlined by Cieślak et al.,^42^ aiming to discover new monoamine oxidase inhibitors solely with the tools available in Qsarna.

We began by uploading all compounds from ChEMBL^55^ that had assigned *K*_i_ or IC_50_ values for either MAO-A or MAO-B assays. Qsarna then preprocessed these data automatically, removing duplicates and salt counterions, where the ligand is neutralized. We made one modification to the protocol by converting the regression problem into a binary classification problem, designating the positive class to all compounds with *K*_i_ or IC_50_ below 100 nM. This allowed us to integrate the results from both functional and binding assays. Additionally, we combine activity measurements from multiple organisms for which the amino acid composition of the binding pocket is conserved. Machine learning models were trained separately for the MAO-A and MAO-B targets, achieving 0.92 ROC AUC for MAO-A and 0.88 ROC AUC for MAO-B. The ROC curves are presented in Supporting Information A.

Subsequently, the top three machine learning models screened the MolPort catalog of available compounds. Only 556 compounds were retained by applying a classification threshold greater than 0.65. Each of these compounds was preprocessed in Qsarna and docked to the MAO-A and MAO-B structures sourced from PDB^56^ (PDB IDs: 2BXR^57^ and 2V5Z,^58^ respectively), and the final selection of molecules relied on visual inspection of the binding poses and consideration of the economic factor.

Finally, the selected group of 19 compounds was acquired and evaluated for MAO-A and MAO-B activities with the Merck inhibitor screening kit (MAK295 and MAK296), which led to the discovery of more potent binders than those reported in the original study by Cieślak et al.^42^ The most effective compound reached 1.37 nM IC_50_ for MAO-B. The multistep filtering pipeline and most potent MAO-B inhibitors are displayed in Figure 2. The experimental details for determining IC_50_ are presented in Supporting Information B.

**Figure 2:**
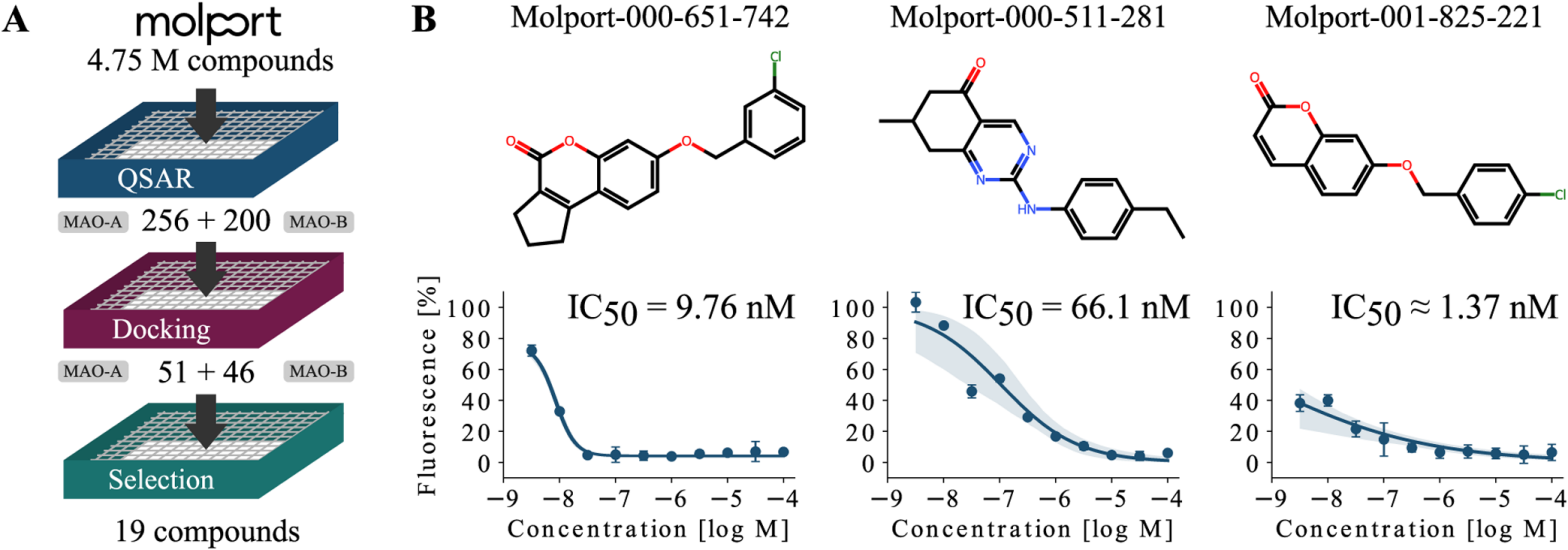
Results of the MAO inhibitor case study. (A) A large MolPort library was screened using the QSAR models trained on ChEMBL data and then further filtered by molecular docking and visual inspection. (B) The three most potent MAO-B inhibitors identified in biochemical assays and their dose–response curves are shown.

## Conclusions

We present Qsarna, an online platform designed to automate the search for novel bioactive compounds in a virtual screening campaign by effectively navigating the chemical space. This tool allows users to manage their data and share experimental findings with team members. The platform features automated machine learning and molecular docking screening tools. Moreover, the fragment-based module provides generative models for developing combinatorial libraries. The platform’s hybrid approach is particularly valuable for targets with limited experimental data, hit expansion campaigns, and reducing chemical bias through the integration of both ligand- and structure-based methodologies. We believe that Qsarna will expedite drug discovery efforts by automating computational screening procedures. Additionally, the user-friendly interface can support experiments and guide scientists who are not strongly familiar with in silico drug design procedures.

## Supplementary Material





## Data Availability

Qsarna is available online to all users at https://qsarna.com. Every user has a monthly limit on the number of compounds analyzed and calculations performed, as the service is provided free of charge with limited resources. For academic projects, we are open to increasing these limits upon request.

## Notes

The raw data for all compounds tested in biochemical assays containing SMILES strings, percentage inhibition at three concentrations, and IC_50_ for the secondary screen (XLSX)
Section A includes the ROC curves of the top three models trained to predict MAO inhibition; Section B includes the experimental details for IC_50_ determination; Section C presents the results of the decoy-based virtual screening evaluation; Section D includes a comparison of available web-based ADMET tools; Section E depicts the software architecture; Section F shows the graphical user interface; and Section G includes the evaluation of the generative fragment-linking module (PDF)
