# Supplementary material for: Qsarna: An Online Tool for Smart Chemical Space Navigation in Drug Design
Source: J Chem Inf Model. 2025 Jul 30;65(15):7811–6. doi: 10.1021/acs.jcim.5c00720 (PMC12344758; doi:10.1021/acs.jcim.5c00720)
Supplement: Supplementary file 2 [file ci5c00720_si_002.pdf]

Supporting Information for
Qsarna: an online tool for smart chemical space navigation in drug design

Marcin Cieślak,[*,†,‡,¶] Jan Łęski,[¶] Olga Krzysztyńska-Kuleta,[§] Justyna Kalinowska-Tłuścik,[¶] and Tomasz Danel[*,¶]

†*Chemistry Department, Selvita, Kraków, Poland*

‡*Doctoral School of Exact and Natural Sciences, Jagiellonian University, Kraków, Poland*

¶*Faculty of Chemistry, Jagiellonian University, Kraków, Poland.*

§*Cell and Molecular Biology Department, Selvita, Kraków, Poland*

E-mail: marcin.cieslak@doctoral.uj.edu.pl; tomasz.danel@uj.edu.pl

A. Case study model performance

For each MAO variant, MAO-A and MAO-B, distinct QSAR models were developed, combining various machine learning techniques (RF, SVM, ANN) with different molecular representations. Each model was evaluated on a test dataset. For each MAO variant, the three best-performing models were selected, and their ROC AUC curves were presented in Figure S1.

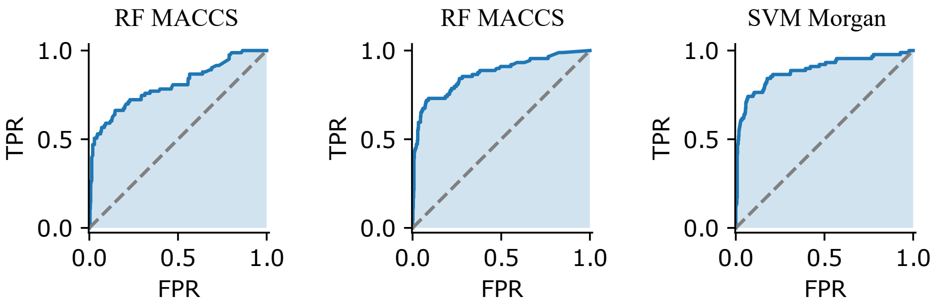

(a) MAO-A

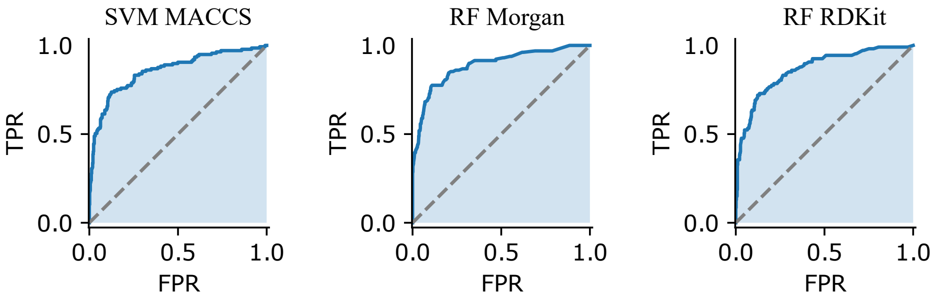

(b) MAO-B

Figure S1: Receiver operating characteristic (ROC) curves computed on the testing set for the top three MAO-A and MAO-B models.

B. Assay procedure

To evaluate the IC_{50} of inhibitors, two fluorometric assays were used: the Monoamine Oxidase-A Inhibitor Screening Kit and the Monoamine Oxidase-B Inhibitor Screening Kit (Merck), according to the manufacturer's protocol. The Echo dose-response software was used to prepare the plates. The compounds were tested at 10 concentrations, starting from 100 µM with a dilution factor of 3.16. All compounds were tested in duplicate in a final concentration of 1% of DMSO. Using a Mantis Liquid Dispenser (Formulatrix), 12.5 µL of protein was added to each tested compound (final concentration of 56 nM) and incubated for 60 minutes at 25°C (MAO-A) or 60 minutes at 37°C (MAO-B). Subsequently, the enzymatic reaction was initiated by adding 10 µL of substrate per well, followed by incubation for 60 minutes at 25°C (MAO-A) or 60 minutes at 37°C (MAO-B). The fluorescence intensity was measured using a plate reader (BioTek Synergy H1) with the following settings: excitation at 535 nm and emission at 587 nm. The data were normalized against low control (assay buffer

containing substrate) and high control (protein and substrate). The results were presented as a percentage of inhibition.

C. Virtual screening decoy benchmark

To evaluate the virtual screening capabilities of the Qsarna platform, decoy datasets were constructed for three structurally diverse protein targets: cyclin-dependent kinase 2 (CDK2), dopamine receptor D3 (DRD3), and monoamine oxidase B (MAO-B). Bioactivity data for each target were retrieved from the ChEMBL database and subsequently partitioned into training and testing subsets using scaffold-based splitting methodology. This approach ensures that the chemical scaffolds present in the testing set remain structurally distinct from those in the training compounds. All compounds with $IC_{50} < 100\,nM$ were considered active. Decoy compounds corresponding to the active molecules in each testing set were generated following the Directory of Useful Decoys-Enhanced (DUD-E) protocol. The decoy libraries were obtained from the DUD-E repository, with a selection criterion of 50 decoys per active compound. Decoy selection was based on physicochemical similarity matching with respect to calculated lipophilicity (cLogP), molecular weight, hydrogen bond donor and acceptor counts, and rotatable bond counts, consistent with established DUD-E methodology.

Comparative docking studies were performed using co-crystal structures retrieved from the Protein Data Bank: 2WXV for CDK2, 7CMV for DRD3, and 2V5Z for MAO-B. Glide docking calculations were executed using Schrödinger Suite v14.1.138, incorporating their ligand preparation workflow. Smina docking was performed within the Qsarna framework, utilizing an automated ligand preparation pipeline that encompasses geometry optimization, tautomer enumeration, and protonation state assignment. An additional variant included stereoisomer generation to assess the impact of stereochemical diversity. Furthermore, machine learning-based quantitative structure-activity relationship (QSAR) models were developed using Qsarna's automated ML module, trained on the active and inactive compound

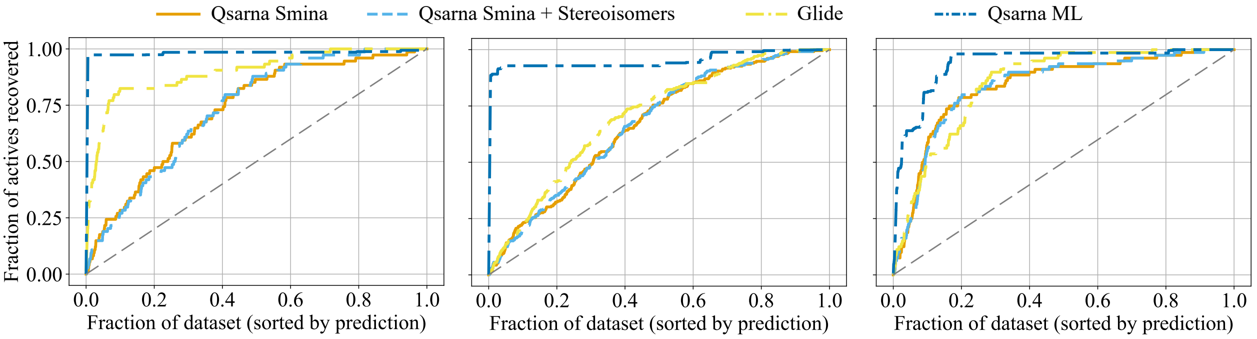

(a) CDK2 (b) DRD3 (c) MAO-B

Figure S2: Enrichment curves plotted for docking and ML methods in the decoys experiment for three protein targets. Qsarna Smina is the Smina algorithm run with the ligands prepared in Qsarna.

datasets from ChEMBL. The enrichment curves of these algorithms are presented in Figure S2.

In the comparison of the results on the three selected targets, Qsarna ML models outperform classical docking algorithms. However, this apparent advantage may be attributable to inherent bias in the decoy benchmark methodology, as the generated decoys contain structural motifs distinguishable from active compounds by ML algorithms. Conversely, active compounds may retain structural similarities despite scaffold-based dataset partitioning, potentially favoring ML-based approaches over physics-based docking methods. Smina algorithm with ligand preparation implemented in Qsarna gives similar results for the versions with and without generated stereoisomers. This may be caused by a small number of undefined stereocenters in the active compounds sourced from ChEMBL. When benchmarked against the commercial Glide software, Qsarna's docking performance was comparable for DRD3 and MAO-B targets, with Smina exhibiting marginal superiority in the MAO-B evaluation. However, Glide demonstrated significantly enhanced performance in the CDK2 virtual screening experiment

D. Performance of ADMET models

The ADMET models available in Qsarna were trained using the same automatic ML module that the users can use to train their QSAR models. The datasets sourced from TDC were split automatically into training and testing subsets using the scaffold-based splitting methodology. The automatically selected model was evaluated on the testing set, which was also uploaded to the publicly available ADMET platforms, ADMETlab 3.0 and admetSAR 3.0, to compare their performance. The results of the evaluation are presented in Figure S3.

Our models (hERG, CACO-2, and logD) demonstrated superior predictive performance, and for BBB permeability, performance was comparable. However, since the training datasets used by ADMETLab and admetSAR are not publicly available, it is unclear whether there is any overlap between their data and ours. It is also possible that our test set may contain identical compounds as those included in the training sets of ADMETlab 3.0 or admetSAR 3.0. This could have artificially improved their performance. Therefore, the results should be interpreted with appropriate caution.

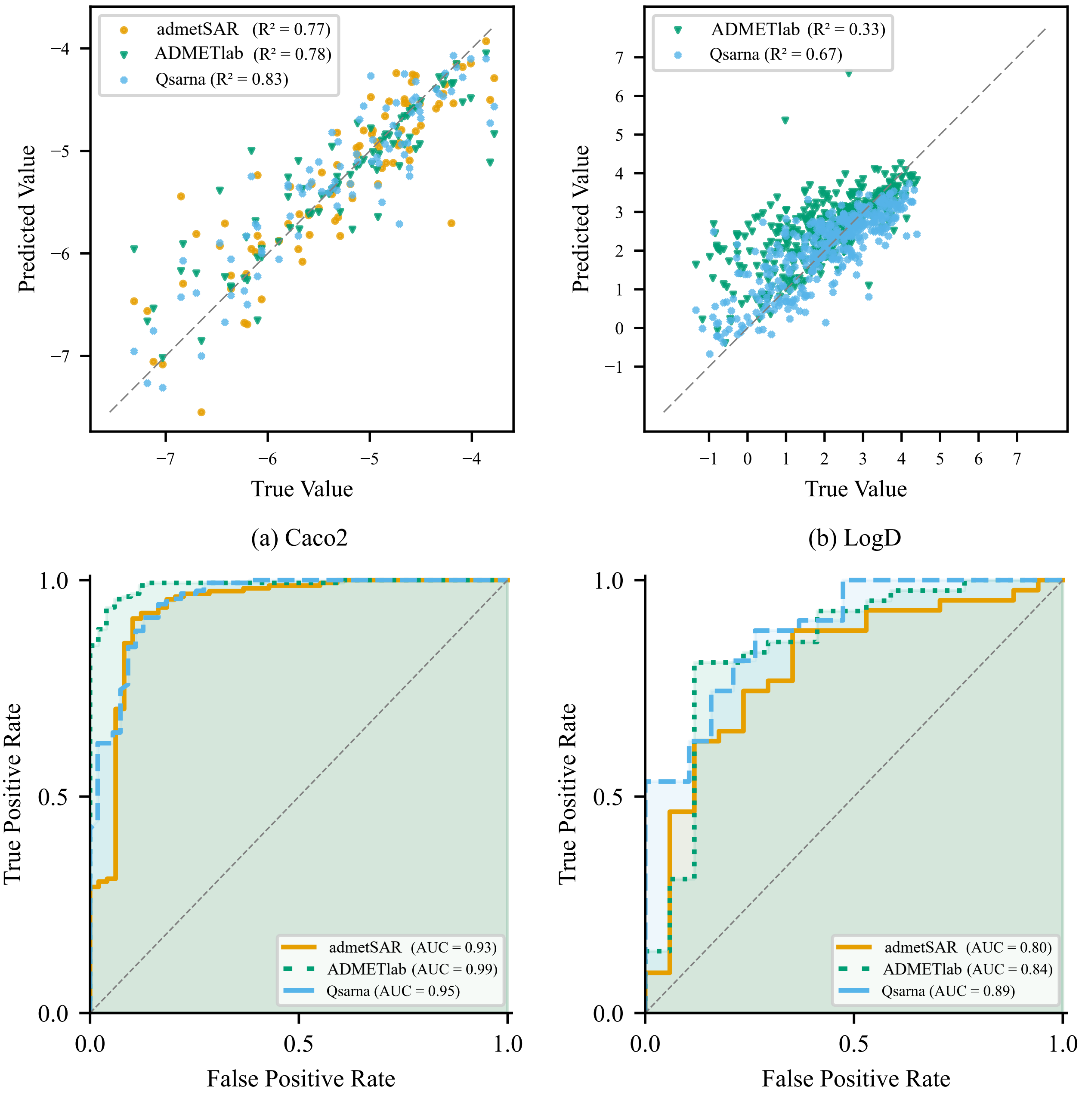

(a) Caco2

(b) LogD

(c) BBBP

(d) hERG

Figure S3: Comparison of ADMET models for two continuous properties, Caco2 and LogD, and two binary properties, blood-brain bareer permeability (BBBP) and hERG inhibition.

E. Software architecture

Qsarna runs as a scalable web service on Amazon Web Services (AWS) for large-scale collaborative analyses, or locally via Docker for institutions requiring data privacy or offline access. Figure S4 shows both architectural configurations. The web version is available for free at `https://qsarna.com`. The containerized solution may be licensed upon request.

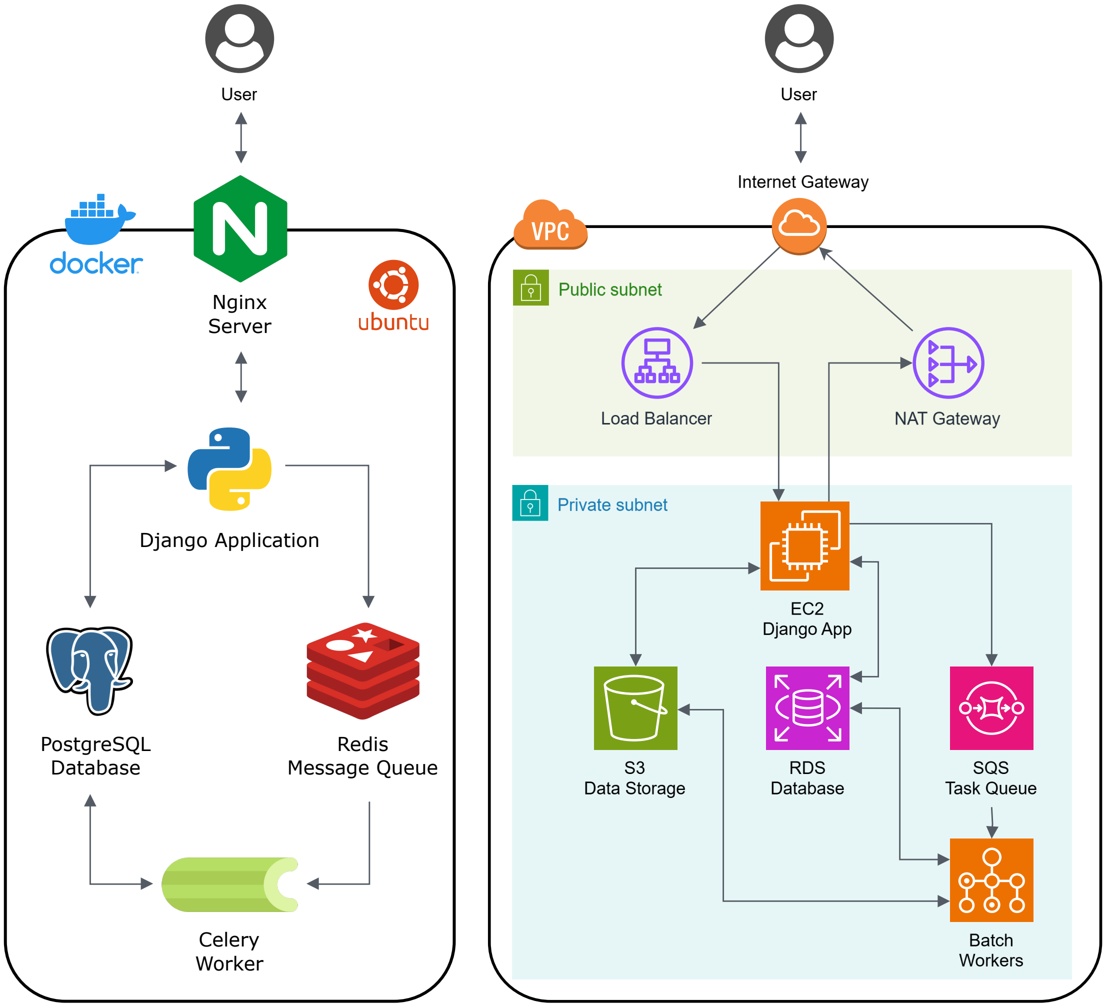

(a) Docker container (b) AWS architecture

Figure S4: Qsarna architecture deployment options. **(a)** Local deployment utilizes a containerized environment with an Ubuntu Docker image that integrates a Django web framework, PostgreSQL database for data persistence, and Redis with Celery for distributed task processing. **(b)** Cloud deployment leverages AWS within a secure virtual private cloud (VPC), utilizing managed services including RDS for database operations, S3 for scalable data storage, EC2 for web application hosting, and SQS/Batch for distributed computational workloads.

F. Graphical Overview of the Web Platform

The application interface has been carefully designed to be intuitive and user-friendly. The clearly structured menu, segmented into dedicated functional modules, is presented in Figure S5.

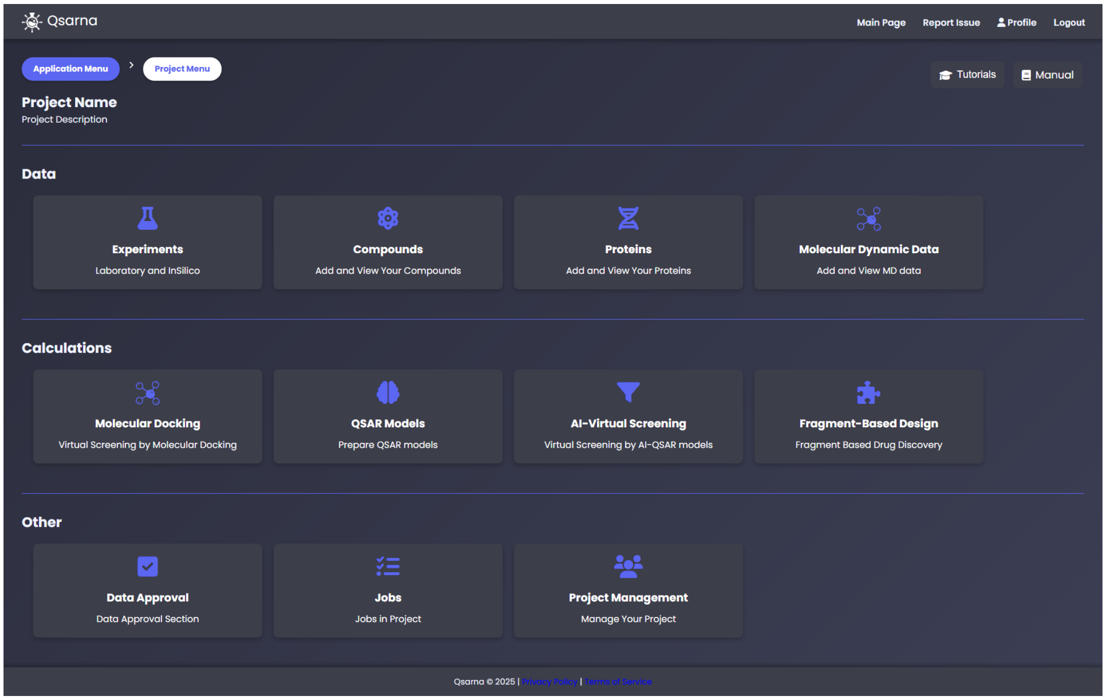

Figure S5: The Project Menu interface, showing access to key modules and user tools.

In the application, individual operations—such as computations or data submissions—are executed through dedicated input forms. An example form for uploading compounds to the database is shown in Figure S6.

The platform facilitates unified data storage and offers an intuitive, summary view for managing and reviewing specific information (Figure S7).

The application also provides a dedicated molecular docking viewer, offering a clear visualization of docking poses along with a table of docking scores and the ability to annotate results with user comments (Figure S8).

A dedicated view displays a performance plot of the QSAR model alongside key evaluation metrics, providing insight into the model's accuracy and robustness (Figure S9).

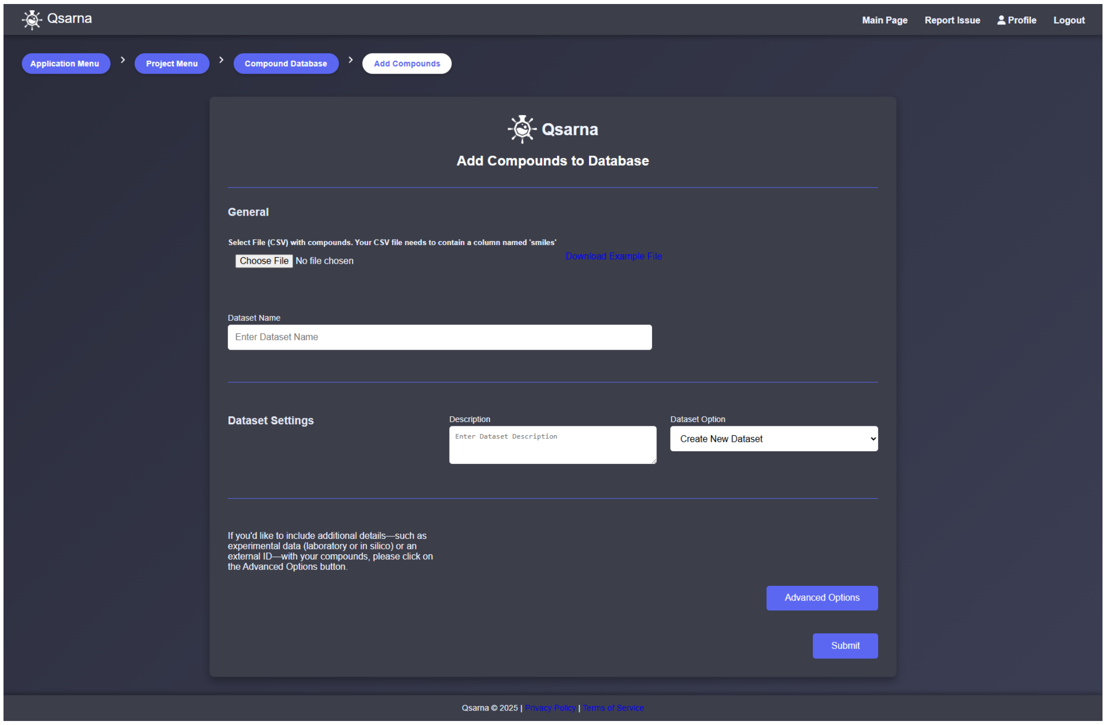

Figure S6: Submission form for uploading compounds to an existing or new dataset in the system.

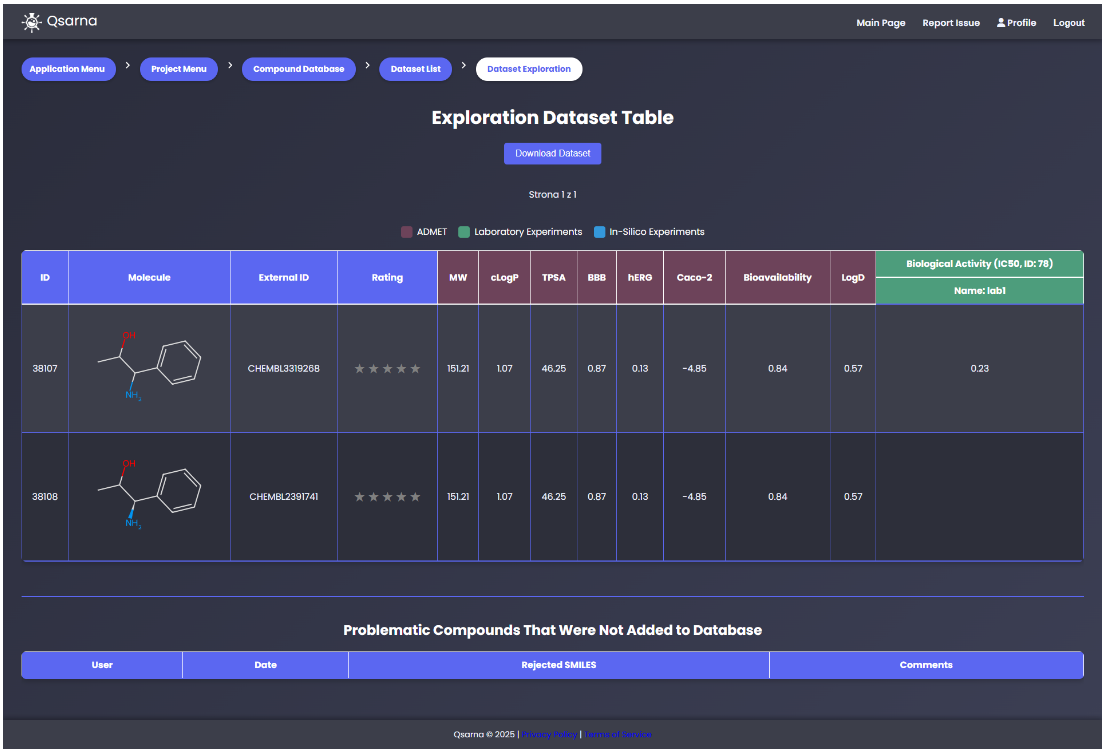

Figure S7: Dataset view designed to support streamlined data analysis and decision-making.

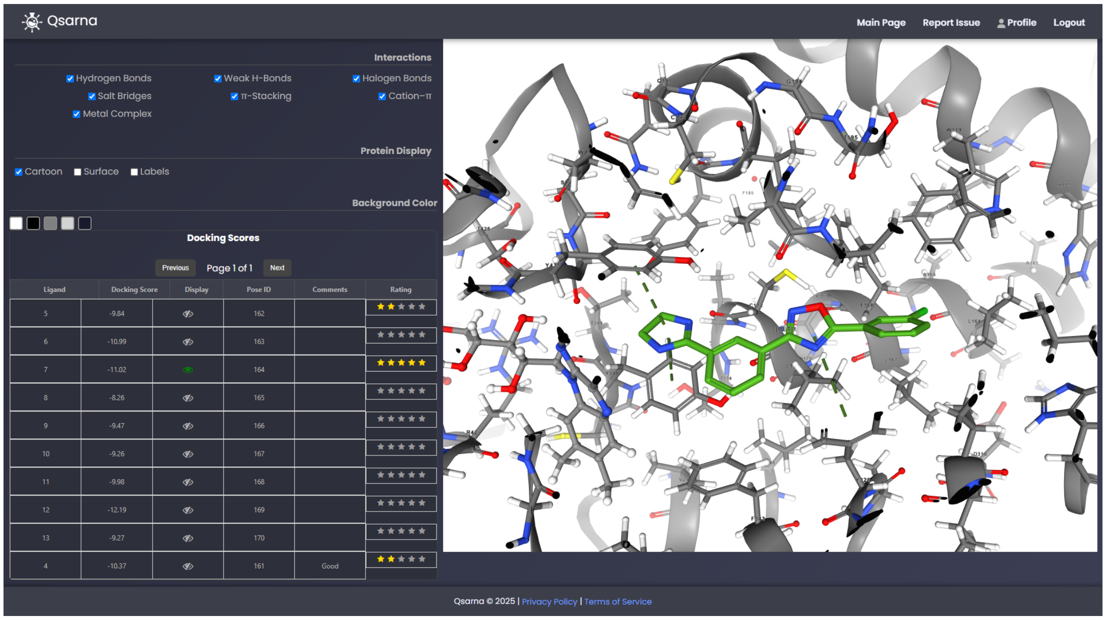

Figure S8: Molecular docking viewer showing visualized poses, docking scores, and a comment section for result interpretation.

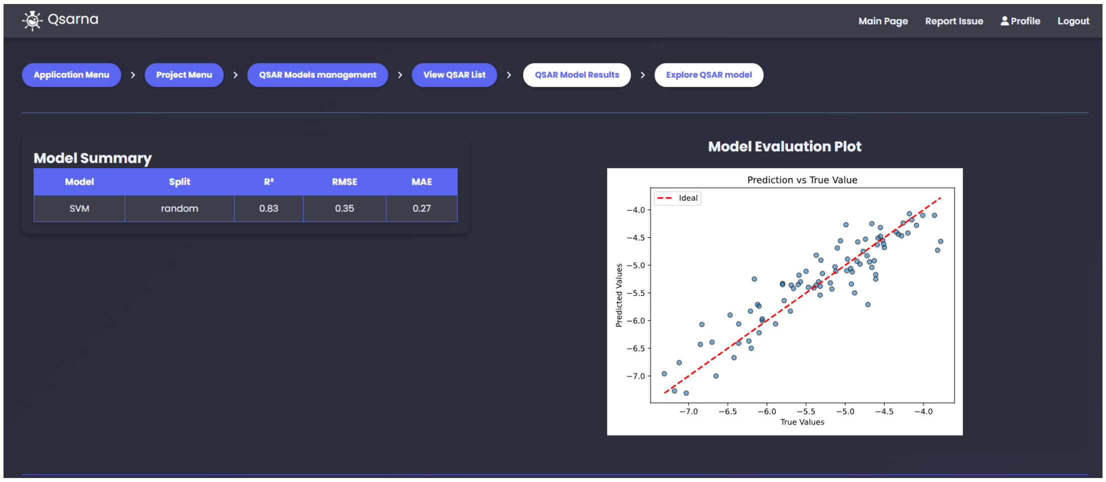

Figure S9: QSAR model quality overview with performance plot and key metrics.

G. Evaluation of the fragment linking module

To demonstrate the usefulness of our fragment-based generative module, we created a benchmark in which 100 fragment pairs were docked into the binding pockets of the MAO-B (PDB ID: 2V5Z) and 5-HT_{2A} (PDB ID: 6A93) proteins. The fragments were sourced from the Enamine fragment library. We compared our model implemented in Qsarna, CRET, with other deep-learning models. The results are shown in Table S1. DeLinker produces more valid linkers, but it does not provide 3D information about the predicted binding pose. In contrast, both DiffLinker and CRET generate linkers within the binding pocket, with CRET producing a larger number of unique and synthetically accessible molecules. Detailed evaluation can be found in the publication about the CRET algorithm[1].

Table S1: Comparison of linking methods for two protein targets (5-HT2 and MAO-B) in terms of the number of unique linkers (maximum 5 attempts), percentage of successfully linked fragments, Tanimoto diversity, synthetic accessibility (SA), and drug-likeness (QED).

**Target**	**Method**	**Unique**	**Valid (%)**	**Diversity**	**SA**	**QED**
5-HT_{2A}	DiffLinker	3.04 ± 1.62	95%	0.54 ± 0.10	4.12 ± 0.59	0.55 ± 0.10
	DeLinker	4.72 ± 1.09	95%	0.47 ± 0.06	3.89 ± 0.53	0.48 ± 0.11
	CRET	3.30 ± 2.13	75%	0.52 ± 0.08	3.94 ± 0.68	0.50 ± 0.11
MAO-B	DiffLinker	2.43 ± 1.76	89%	0.58 ± 0.10	4.46 ± 0.76	0.57 ± 0.11
	DeLinker	4.61 ± 1.27	93%	0.46 ± 0.05	3.83 ± 0.50	0.55 ± 0.12
	CRET	3.06 ± 2.03	76%	0.51 ± 0.09	3.89 ± 0.72	0.59 ± 0.11

References

(1) Cieślak, M.; Danel, T.; Kalinowska-Tłuścik, J. Structure-guided fragment linking algorithm enables chemically feasible ligand design with predicted binding modes. *ChemRxiv* **2025**, doi:10.26434/chemrxiv-2025-jz8d3 This content is a preprint and has not been peer-reviewed.
